# Efficacy of Thalidomide Treatment in Children With Transfusion Dependent β-Thalassemia: A Retrospective Clinical Study

**DOI:** 10.3389/fphar.2021.722502

**Published:** 2021-08-12

**Authors:** Xinyu Li, Shuting Hu, Yong Liu, Junjiu Huang, Weicong Hong, Luhong Xu, Honggui Xu, Jianpei Fang

**Affiliations:** ^1^ Guangdong Provincial Key Laboratory of Malignant Tumor Epigenetics and Gene Regulation, Sun Yat-sen Memorial Hospital, Sun Yat-sen University, Guangzhou, China; ^2^ Department of Pediatrics, Sun Yat-sen Memorial Hospital, Sun Yat-sen University, Guangzhou, China; ^3^ Children’s Hospital of Shanghai, Shanghai Jiaotong University, Shanghai, China; ^4^ MOE Key Laboratory of Gene Function and Regulation, State Key Laboratory of Biocontrol, School of Life Sciences, Sun Yat-sen University, Guangzhou, China

**Keywords:** efficacy, safety, transfusion dependent β-Thalassemia, thalidomide, fetal hemoglobin

## Abstract

**Background:** Thalidomide has been reported as a promising treatment for reducing transfusion volume in adults with β-thalassemia. However, the evidence about the safety and efficacy of thalidomide on children with transfusion dependent β-thalassemia (TDT) is scarce.

**Methods:** Seventy-seven children with TDT treated with thalidomide at least for 6 months were included and retrospectively analyzed. Oral dose was started at 2.5 mg·kg-1·d-1. Blood volume for maintenance of hemoglobin above 90 g·L-1 compared with pre-treatment volume is the evaluation index for response.

**Results:** After the sixth month treatment, 51/77 (66.2%) maintained Hb over 90 g·L-1 without transfusion. Adverse events were reported in 48 (63.2%) patients. Age, sex, genotype category, dosage, and transfusion interval before thalidomide treatment were not correlated to treatment response. The AUC was 0.806 for the HbF at the third month of treatment in predicting probability of major responders at the sixth month treatment. Based on Youden’s index algorithm in the ROC curve, 47.298 g·L-1 was the optimal cut-off value of the HbF at the third month of treatment in predicting major responders at the sixth month treatment, with sensitivity of 67.5% and specificity of 93.3%.

**Conclusions:** The dose of thalidomide between 2.5 mg·kg-1·d-1 and 3.6 mg·kg-1·d-1 is effective in TDT children. Severe side effects are uncommon. HbF concentration of 47.298 g·L-1 at the third month is recommended as the predictor for further major responders.

## Background

Thalassemia is one of the most common single gene inherited hematopoietic disorder (Taher et al., 2019). In β-thalassemia, hemoglobin (Hb) production is insufficient because of mutations or deletions of *HBB* gene, causing an imbalance between α-globin and β-globin. Complete deficiency of β-globin is regarded as β^0^ and partial β-globin deficiency is regarded as β^+^. According to the requirement of transfusion, β-thalassemia is classified into non-transfusion-dependent β-thalassemia (NTDT) (transfusions required under certain conditions such as infection, pregnancy or surgery) and transfusion-dependent β-thalassemia (TDT) (regular and lifelong transfusions required) (Barnett, 2019). Patients with β-hemoglobinopathies with higher fetal hemoglobin (HbF) level present with milder phenotypes than those with lower HbF level.

Inducers of HbF, such as hydroxyurea and histone deacetylase inhibitors, are effective in improving hemoglobin level in some patients with β-thalassemia (Ren et al., 2018; Shah et al., 2020). Thalidomide is an immunomodulatory agent with antiangiogenic properties. Clinical studies have demonstrated that thalidomide exerts promising effects on ameliorating anemia in patients with myeloproliferative disorders, sickle cell disease and β-thalassemia (Musto et al., 2002; Aerbajinai et al., 2007; Chen et al., 2017; Shah et al., 2020). Previous studies have also shown that thalidomide is effective in some adult patients with β-thalassemia (Masera et al., 2010; Chen et al., 2017; Yassin, 2020), but still not recommended to children for scarce evidence of safety and efficacy. Therefore, we performed this retrospective study to provide more evidence for the use of thalidomide in children with TDT β-thalassemia. Furthermore, we evaluated the potential predictors for response to thalidomide in children with TDT.

## Methods

### Patients

This retrospective study included patients with TDT accepted thalidomide in Department of Pediatrics, Sun Yat-sen Memorial Hospital between March 2018 and December 2019. They have been followed up at least for 6 months since the application of thalidomide. All patients were diagnosed according to the clinical characteristics and genetic test. Clinical data were obtained from patients’ medical records and the hospital electronic database. Those who did not have transfusion volume record before and during thalidomide treatment, or who did not have first month, third month visit were excluded from efficacy and safety analysis. The included patients did not have history of hydroxyurea (Hu) treatment. Neither did they have Hu prescribed during the application of thalidomide.

The application of thalidomide to the patients was approved by the off-label medicine committee. The retrospective study protocol was approved by the Ethics Committee of Sun Yat-sen Memorial Hospital and was in accordance with the 1964 Declaration of Helsinki and its later amendments. The clinical trial registration number is ChiCTR2000031436.

### Diagnosis

TDT entails lifelong regular transfusion requirement for survival when the patients were confirmed diagnosed with complexed heterozygote or homozygote of β-globin gene mutations. Complete deficiency of β-globin expression is regarded as β^0^ and partial β-globin expression deficiency is regarded as β^+^. β^0^/β^+^, β^0^/β^0^, and β^+^/β^+^ are representing complexities of β^0^ globin genes and/or β^+^ globin genes.

### Treatment

#### Thalidomide

Thalidomide was produced by Suzhou Changzheng-Xinkai Pharmaceutical Co. Ltd. (China). Oral dose was started at 2.5 mg·kg-1·d-1. And dose was adjusted based on the response to thalidomide and the occurrence of adverse effects at third month visit. The maximum dose was 4.0 mg·kg-1·d-1 under the guidance from the off-label medicine committee.

#### Transfusion and Iron Chelation

Transfusion was recommended to maintain Hb higher than 90.0 g·L-1 during treatment. If Hb level decresed to lower than 90.0 g·L-1, regular volume of transfusion should be enforced. Hb level below 80.0 g·L-1 was not allowed. Iron chelation therapy was continued if serum ferritin (SF) was above 1,000 ng/ml during treatment. One or two of deferiprone, deferoxamine and deferasirox were recommended iron chelating agents according to the SF level.

### Treatment Response Criteria

For patients with TDT β-thalassemia, maintenance of hemoglobin above 90 g·L-1 without blood transfusion after treatment was defined as a major response; and maintenance of hemoglobin above 90 g·L-1 with 50% or over 50% reduction of transfusion volume was defined as a minor response; otherwise, the treatment was considered ineffective. All laboratory parameters of TDT patients were examined 2 weeks after the last transfusion. Baseline Hb level was referred to the last test measured before thalidomide treatment.

### Toxicity Evaluation

The occurrence of toxicity was retrospectively collected according to history record and laboratory test record. Suspected related adverse events including lethargy, arthralgia, edema, headache, vomiting, nausea, stomachache, dizziness, constipation, diarrhea, distension of the abdomen, peripheral neuropathy (numbness of extremities), chest tightness, and skin rash, were recorded. White blood cell counts, absolute neutrophil count, platelet counts, aspartate aminotransferase levels, alanine aminotransferase levels, total bilirubin levels, γ-glutamyl transpeptidase, creatine levels, and blood urea nitrogen levels were also recorded. Adverse events and toxicity were graded by modified Common Terminology Criteria for Adverse Events (CTCAE) v5.0. According to the guidance from off-label medicine committee, reduced dose or withdrawal thalidomide was performed if grade III/IV adverse events occurred.

### Supportive Care

During treatment, patients received supportive treatments to alleviate symptoms of adverse effects, including adenosine triphosphate for lethargy, fiber rich diet for constipation, vitamin D for arthralgia, vitamin B6 for numbness, anti-allergic agents for rash, and liver protecting agents. Aspirin were prescribed to patients with platelets >500 × 10^9^/L to prevent thrombosis. No prophylatic anti-thrombosis agent was applied.

### Statistical Analysis

All patients follow-up was done by outpatient service and telephone. All patients without outpatient follow-up records within 6 months were confirmed by telephone follow-up. Follow-up was updated as of 30th September 2020. Complete blood counts, Hb composition analysis, transfusion volume were compared before and after treatment. Chi square tests were used to calculate comparisons between groups. Data were shown as mean (95% confidence interval) or median (range). Changes in HbF level were compared using Kruskal-Wallis H test between different groups of treatment response. One-way ANOVA analysis of variance or Kruskal-Wallis H test was performed to determine factors that predicted efficacy, including start dosage of thalidomide, thalidomide dosage after third month visit and age. Cochran-Mantel-Haenszel Chi-square test was performed to compare the distribution of variants of sex, genotype category, and transfusion interval before thalidomide treatment, whether Hb decreased during the first month of treatment between different groups of treatment response. To evaluate the performance of independent risk factors in predicting major responders, the receiver-operating characteristic (ROC) curve analysis was performed and the optimal cut-off values were obtained from the Youden’s index. Diagnostic accuracy was expressed as the sensitivity, specificity, positive predictive value, negative predictive value, and the area under the ROC curve (AUC). Statistical analysis was performed by using SPSS 22.0 (SPSS Institute, Cary, NC). *p* values < 0.05 were considered significant.

## Results

### Patient Characteristics

There were 112 patients accepted thalidomide treatment in Department of Pediatrics, Sun Yat-sen Memorial Hospital between March 2018 and December 2019. There were 19 patients who did not have transfusion volume record before and during thalidomide treatment, while 10 patients did not have the first month visit, and six patients did not have the third month visit. Finally, 35 patients were excluded from efficacy and safety analysis for thalidomide. The 77 included patients’ clinical characteristics were illustrated in Table 1.

**TABLE 1:** The patients’ clinical characteristics (*n* = 77).

Characteristics	
Age, median [range] (years)	10 [5,18]
Sex	
Male, *n* (%)	45 (58.4%)
Female, *n* (%)	32 (41.6%)
Start dosage of Thalidomide, mean (95%CI) [g/(kg·d)]	2.62 (2.42, 2.82)
Dosage of Thalidomide after third month visit, mean (95%CI) [g/(kg·d)]	3.61 (3.36, 3.85)
Genotype category	
β^0^/β^0^, *n* (%)	21 (27.3%)
β^0^/β^+^, *n* (%)	29 (37.7%)
β^+^/β^+^, *n* (%)	27 (35.0%)
Transfusion volume before Thalidomide treatment, mean (95%CI) [ml/(kg.month)]	23.55 (21.32, 25.79)
Transfusion interval	
≥28 days, *n* (%)	18 (23.4%)
<28 days, *n* (%)	59 (76.6%)
Combined with AIHA, *n* (%)	3 (3.9%)
Splenectomy before, *n* (%)	3 (3.9%)
Baseline HbF, mean (95%CI) (g/L)	8.44 (6.72, 10.17)

a Abbreviation: AIHA, autoimmune hemolytic anemia; HbF, fetal hemoglobin.

Complete deficiency of β-globin is regarded as β^0^ and partial β-globin deficiency is regarded as β^+^.

### Treatment Outcomes in Patients With Transfusion-Dependent β-Thalassemia

**FIGURE 1:**
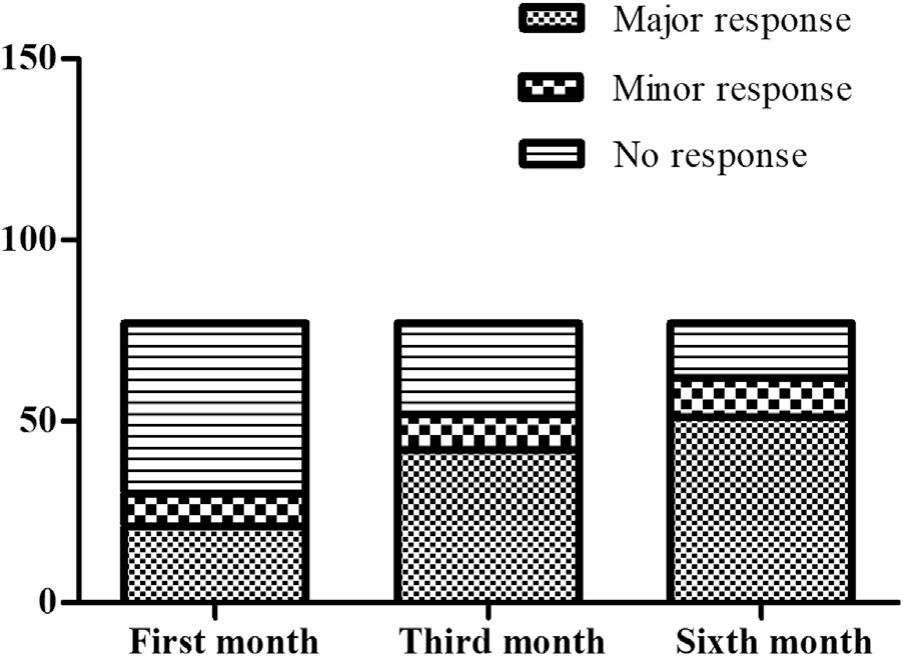
The comparison for the responder composition at the first month, the third month and the sixth month. At the first month evaluation, there were 21 major responders, nine minor responder and 47 non-responders. At the third month evaluation, there were 42 major responders, 10 minor responder and 25 non-responders. At the sixth month evaluation, there were 51 major responders, 11 minor responder and 15 non-responders.

The treatment outcomes in the patients treated with thalidomide were evaluated by transfusion volume at the first month, the third month and the sixth month visits when Hb was kept above 90.0 g·L-1. The results of evaluation are illustrated as Figure 1. After the first month treatment, 21/77 (27.3%) maintained Hb over 90 g·L-1 without transfusion. After the third month treatment, 42/77 (54.5%) maintained Hb over 90 g·L-1 without transfusion. After the sixth month treatment, 51/77 (66.2%) maintained Hb over 90 g·L-1 without transfusion. When it came to the transfusion interval, 49 patients terminated transfusion with Hb over 90 g·L-1, while 23 patients presented prolonged transfusion interval by the end of the sixth month treatment, comparing with baseline. Five patients consisted with regular transfusion without decreased volume or prolonged interval.

### Analysis of Parameters That May Affect Efficacy

#### Dosage and Efficacy

The dosage for deferent response types at the first month, the third month and the sixth month were compared separately (as shown in Table 2). The median dosage from the beginning to the third month was 2.5 mg·kg-1·d-1. Dosage was adjusted at the third month visit according the first month and third month response evaluation or adverse events. The median dosage from the third month to the sixth month was 3.6 mg·kg-1·d-1. When the response rate at the sixth month evaluation was compared with the third month evaluation, there were six patients accepted previous dosage, 32 accepted higher dosage and seven accepted lower dosage among those whose response was improved. However, there were six patients accepted previous dosage, 20 accepted higher dosage and six accepted lower dosage among those whose response was not improved. The proportion for improvement in response with higher dose (32/45) and the proportion for no response improvement with higher dose (20/32) were not significantly different (*p* = 0.715).

**TABLE 2:** The analysis of parameters for response differences at the first month, the third month and the sixth month.

	The first month				The third month				The sixth month			
	Major response (*n* = 21)	Minor response (*n* = 9)	No response (*n* = 47)	*p* value	Major response (*n* = 42)	Minor response (*n* = 10)	No response (*n* = 25)	*p* Value	Major response (*n* = 51)	Minor response (*n* = 11)	No response (*n* = 15)	*p* Value
Age, (years)	10.1 (8.23, 11.96)^d^	10.3 (7.84, 12.8)^d^	9.0 (5.17)^e^	0.723^b^	10.1 (8.96, 11.14)^d^	10.7 (8.0, 13.4)^d^	8 (5.15) ^e^	0.330^b^	10.15 (9.18.11.13)^d^	8.91 (6.46, 11.4)^d^	9.07 (7.48.10.65)^d^	0.371^a^
Sex												
Male, *n*	11	6	28	0.742^c^	23	5	17	0.475^c^	30	6	9	0.958^c^
Female, *n*	10	3	19		19	5	8		21	5	6	
Start dosage of Thalidomide, mean (95%CI) [g/(kg·d)]	2.39 (1.99, 2.79)^d^	3.17 (2.27, 4.08)^d^	2.62 (2.38, 2.85)^d^	0.038^a^	2.57 (2.32, 2.82)^d^	2.61 (1.73, 3.49)^d^	2.71 (2.35, 3.07)^d^	0.823^a^	—	—	—	—
Dosage of Thalidomide after third month visit, mean (95%CI) [g/(kg·d)]	—	—	—	—	—	—	—	—	3.64 (3.35, 3.93)^a^	3.51 (2.51, 4.51)^a^	3.41 (2.14, 5.26)^e^	0.913^b^
Genotype category												
β0/β0, *n* (%)	5	3	13	0.945^c^	12	4	5	0.799^c^	12	6	3	0.331^c^
β0/β+, *n* (%)	9	3	17		15	3	11		21	2	6	
β+/β+, *n* (%)	7	3	17		15	3	9		18	3	6	
Transfusion interval												
≥28 days, *n* (%)	8	5	25	0.996^c^	23	2	13	0.819^c^	11	3	4	0.873^c^
<28 days, *n* (%)	10	4	16		14	7	9		40	8	11	
Whether Hb decreased during the first month of treatment, *n* (%)												
Yes, *n* (%)	10	4	6	0.501^c^	14	7	9	0.085^c^	19	6	5	0.342^c^
No, *n* (%)	8	5	25		23	2	13		28	3	7	
Baseline HbF, (g/L)	11.1 (0.87, 77.18)^e^	9.5 (3.58.15.4)^d^	6.3 (0.5, 38.4)^e^	0.063^b^	10.4 (1.7, 77.18)^e^	5.32 (2.99, 7.64)^d^	5.22 (3.3, 7.1)^d^	<0.001^b^	8.87 (0.625, 77.18)^e^	5.4 (2.51, 8.34)^d^	5.18 (2.65, 7.71)^d^	0.007^b^
HbF at the third month of treatment, (g/L)	—	—	—	—	68.4 (9.43, 121.16)^d^	38.75 (26.78, 50.7)^d^	25.7 (20.95, 42.7)^e^	<0.001^b^	63.1 (54.1, 72.1)^d^	32.2 (22.7, 41.7)^d^	29.8 (11.4.48.2)^d^	<0.001^b^
HbF at the sixth month of treatment, (g/L)	—	—	—	—	—	—	—	—	84.2 (77.0, 91.4)^d^	49.3 (26.7, 71.9)^d^	39.7 (21.7, 57.7)^d^	<0.001^b^

a *p* value from one-way ANOVA analysis of variance.

b *p* value from Kruskal-Wallis H test.

c *p* value from Cochran-Mantel-Haenszel Chi-square test.

d Mean (95% confident interval).

e Percentiles, Q_50%_ (range).

#### Other Parameters and Efficacy

**FIGURE 2:**
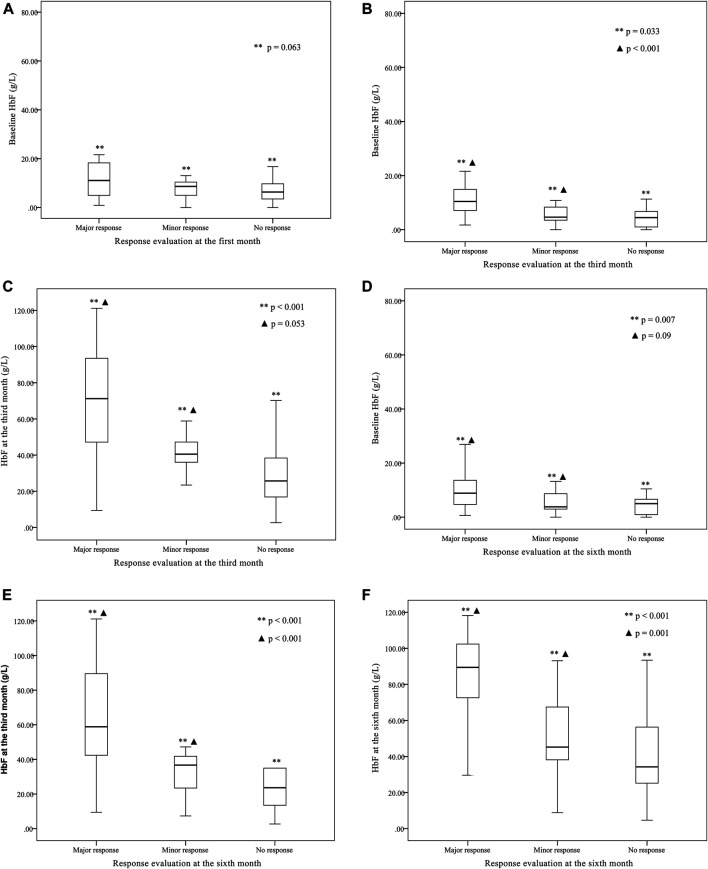
The baseline fetal hemoglobin (HbF) concentration, HbF concentration at the third month of treatment, and HbF concentration at the sixth month of treatment comparison between groups of major response, minor response and no response.**(A)** The baseline HbF concentration were not significantly different between group major response (11.1 (1.7, 77.18) g·L-1), minor response (9.5 (3.58, 15.4) g·L-1) and group no response [6.3 (0.5, 38.4) g·L-1] (**p = 0.063) according to the evaluation at the first month. The differences between any two groups were not significant. **(B)** According to the evaluation at the third month, the baseline HbF concentration was significantly higher in group major response [10.4 (1.7, 77.18) g·L-1] than group minor response [5.32 (2.99, 7.64) g·L-1] and group no response [5.22 (3.3, 7.1) g·L-1] (**p < 0.001, ▲ *p* = 0.033), while the differences between group minor response and group no response were not significant (*p* = 1.000). **(C)** According to the evaluation at the third month, the third month HbF concentration was significantly different between group major response [68.4 (9.43, 121.16) g·L-1], group minor response [38.75 (26.78, 50.7) g·L-1] and group no response [25.7 (20.95, 42.7) g·L-1] (**p < 0.001, ▲ *p* = 0.053), while the differences between group minor response and group no response were not significant (*p* = 0.734). **(D)** According to the evaluation at the sixth month, the baseline HbF concentration was significantly different between group major response [8.87 (0.625, 77.18) g·L-1], group minor response [5.40 (2.51, 8.34) g·L-1] and group no response [5.18 (2.65, 7.71) g·L-1] (**p = 0.007, ▲ *p* = 0.09), while the differences between group minor response and group no response were not significant (*p* = 1.000). **(E)** According to the evaluation at the sixth month, the third month HbF concentration was significantly higher in group major response [63.1 (54.1, 72.1) g·L-1] than group minor response [32.2 (22.7, 41.7) g·L-1] and group no response [29.8 (11.4, 48.2) g·L-1] (**p < 0.001, ▲ *p* < 0.001), while the differences between group minor response and group no response were not significant (*p* = 0.992). **(F)** According to the evaluation at the sixth month, the sixth month HbF concentration was significantly higher in group major response [84.2 (77.0, 91.4) g·L-1] than group minor response [49.3 (26.7, 71.9) g·L-1] and group no response [39.7 (21.7, 57.7) g·L-1] (**p < 0.001, ▲ *p* = 0.001), while the differences between group minor response and group no response were not significant (*p* = 1.000).

Other parameters, including age, sex, genotype category, and transfusion interval before thalidomide treatment, were further analyzed for the differences in response (as shown in Table 2). The baseline HbF concentration, HbF at the third month of treatment, HbF at the sixth month of treatment were significantly higher in group major response than the other two groups, while the differences between minor response group and no response group were not significant (as shown in Figure 2).

### Predictive Values of the HbF for Probability of Responders

**FIGURE 3:**
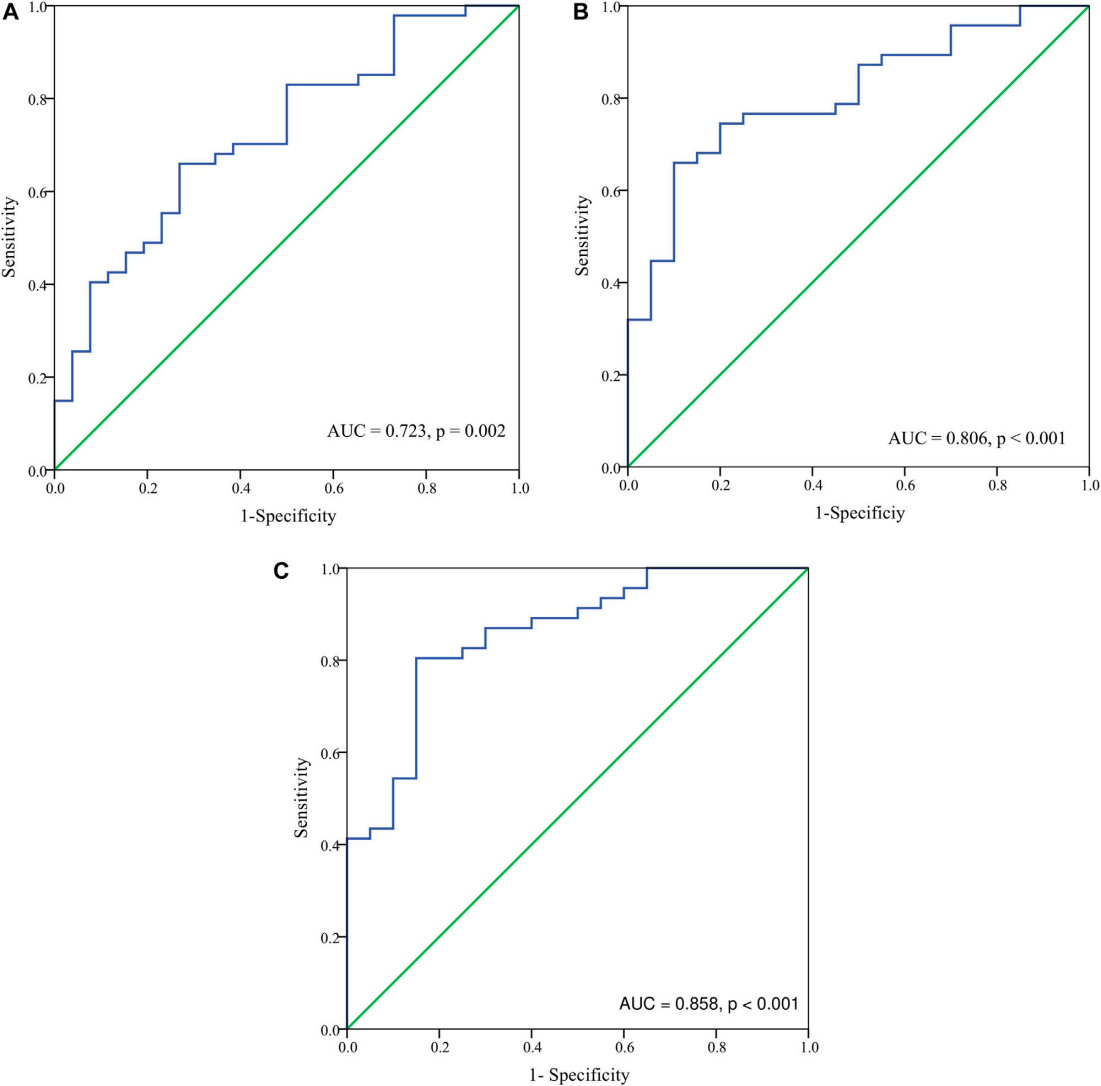
The receiver-operating characteristic (ROC) curve analysis for hemoglobin F (HbF) concentration at different stages in predicting probability of major responders at the sixth month. **(A)** The ROC analysis for HbF concentration at baseline in predicting probability of major responders at the sixth month. The area under the ROC curve (AUC) was 0.723 (*p* = 0.002). **(B)** The ROC analysis for HbF concentration at the third month in predicting probability of major responders at the sixth month. The AUC was 0.806 (*p* < 0.001). **(C)** The ROC analysis for HbF concentration at the sixth month in predicting probability of major responders at the sixth month. The AUC was 0.858 (*p* < 0.001).

Basing on the results of parameters analysis above, we demonstrated the predictive performance of the HbF at different stage in predicting probability of responders by the ROC curves. The AUC was 0.806 for the HbFat the third month of treatmentin predicting probability of major responders at the sixth month treatment (as shown in Figure 3). Based on Youden’s index algorithm in the ROC curve, 47.298 g·L-1 was the optimal cut-off value of theHbFat the third month of treatment in predicting major responders at the sixth month treatment, with sensitivity of 67.5%, specificity of 93.3% (as shown in Table 3).

**TABLE 3:** Best threshold analysis of the HbF concentration at different stages of thalidomide treatment for ROC of major responders by the sixth month treatment.

Test	Best threshold	Specificity	Sensitivity	Accuracy	False positive rate
Baseline HbF, (g/L)	6.915	0.8	0.675	0.723	0.2
HbF at the third month of treatment, (g/L)	47.298	0.933	0.675	0.806	0.067

Abbreviation: HbF, fetal hemoglobin; ROC, receiver-operating characteristic curve.

### Thalidomide-Related Adverse Events

During 6 months follow-up, adverse events were reported in 48 (63.2%) patients, including dizziness and/or lethargy (*n* = 24), constipation (*n* = 9), abdominal pain and/or vomiting and/or nausea (*n* = 9), arthralgia (*n* = 4), edema (*n* = 3), peripheral neuropathy (numbness of extremities) and/or headache (*n* = 2), skin rash (*n* = 1), leukocytopenia (*n* = 7), neutropenia (*n* = 10), thrombocytosis (*n* = 9). Two patients stopped using thalidomide due to adverse events, including central venous thrombosis (*n* = 1) and seizure (*n* = 1). Most the symptoms relieved spontaneously without withdraw, while some symptoms relieved with temporary reduced dosage.

## Discussion

In this study, the safety and efficacy of low dose thalidomide in transfusion-dependent thalassemia children is presented by the retrospective analysis. The most important result of this study is that, the dose of thalidomide between 2.5 mg·kg-1·d-1–3.6 mg·kg-1·d-1 is effective in children with TDT. This is the first available protocol for thalidomide treatment for children with TDT in a large scale. The HbF concentration of 47.298 g·L-1 at the third month of treatment may be the predictor for further treatment response.

The effective dose of thalidomide in *ß*-thalassemia children was not reported in previous studies. In this study, we found that, with the start dose of 2.5 mg·kg-1·d-1, the major response rate and the minor response rate at the third month could be as high as 67.5% (52/77). When the mean dose of thalidomide was increased to 3.6 mg·kg-1·d-1 at the third month to the sixth month, the major response rate and the minor response rate could elevated to 80.5% (62/77) at the sixth month. However, when the proportion for improvement in response with higher dose (32/45) was compared with the proportion for no response improvement with higher dose (20/32), the different was not significant. For this result, the reason for increased response rate cannot be attributed simply to the increased dose.

In previous study, cases were reported that patient with TDT responding to thalidomide effectively increased hemoglobin level for long term with high dose of thalidomide 75 mg·kg-1·d-1 or 100 mg·d-1 (Aguilar-Lopez et al., 2008; Masera et al., 2010), while β thalassemia intermedia was reported responding to low dose of thalidomide (1–2mg·kg-1·d-1) (Fozza et al., 2015). Children cases of β thalassemia major were presented no response to thalidomide (Kalra et al., 2017). The response to thalidomide seemed to be controversial. Phase Ⅱ clinical trial for thalidomide in adult patients with thalassemia intermedia at the dose of 50 mg orally per night presented little data for safety (Li et al., 2018). In Iraqi Kurdistan (Yassin, 2020), a cohort of 37 patients with symptomatic β-thalassemia on the dose of 2–10 mg·kg-1·d-1 achieved response rate of 75.7%, while minimal side effects were documented. The overall response rates of thalidomide (50 mg·d-1 for <30 kg, 100 mg once daily for >30 kg) and hydroxyurea (500 mg·d-1) combination treatmentas 68.2% at 3 months, while thalassaemia intermedia (78.6%) presented higher response rate than thalassaemia major (50%) (Shah et al., 2020), indicating the low response rate in thalassemia major. In a multicenter study in southern China population, the initial dose of thalidomide was 50 mg·d-1, and the dose of 100 mg·d-1 was given to patients needing blood transfusions at least twice a month. The overall response rate was 93.5% (Yang et al., 2020). In the Indian report for 20 patients with transfusion-dependent E-Beta thalassemia (Nag et al., 2020), the starting dose of thalidomide was 50 mg·d-1 in patients less than 12 years age and 100 mg·d-1 in patients more than 12 years age, 15 (71.4%) attained transfusion independence and 1 (4.7%) attained partial response while 5 (23.9%) were non-responders. The major adverse effect documented was constipation (47%).

In this current study, we attained a different approach to dose of thalidomide. It is proposed that the start dose of 2.5 mg·kg-1·d-1 may be suitable and the observation for 3 months for side effects and efficacy would be recommended. For the unclear benefit, the increased dose over 3.6 mg·kg-1·d-1 was not recommended.

In the analysis parameters related to thalidomide efficacy, HbF concentration in peripheral blood before transfusion was the only found parameter that related to the treatment response. According to the ROC analysis results, the HbF concentration prior to response evaluation predicted the response probability. The HbF concentration at the third month may be a dependable predictor for the response at the sixth month. The HbF concentration of 47.298 g·L-1 at the third month could be regarded as the threshold for major responders at the sixth month. Long term observation for more than 6 months is not recommended if there is no evidence for elevated HbF concentration over threshold. However, the HbF concentration is not a good predictor for identification between minor responders and non-responders according to the result that the HbF concentration differences between minor response group and no response group were not significant. In a multicenter adults study (Yang et al., 2020), the ratio of HbF at baseline was an independent risk factor for response to thalidomide in southern China population. The primary response to thalidomide was significantly correlated with the HbF ratio before treatment and splenic status, but not related to age, sex, phenotype (TDT or NTDT), duration of treatment, thalidomide dose or baseline Hb level (Yang et al., 2020). It has been announced that elevated Hb was mainly attributable to increased HbF levels (Yang et al., 2020). For the observation in HbF concentration changes were common in major responders in previous studies, researchers performed gene polymorphisms study. Single nucleotide polymorphisms (SNPs) in HBG2 and HBS1L-MYB were reported to be related to thalidomide response in Chinese patients, while the cumulative number of minor SNP alleles may be good predictors of treatment response (Yang et al., 2020). These results might support good indicators for targeted prescription of thalidomide in Chinese patients, but not necessary good indicators for global promotion, considering genomic diversity between races. HbF concentration may be a general predictor for thalidomide treatment response, which needs further validation. Moreover, the underlying mechanism was yet clarified. Further study of genetic and metabolism reasons for differences in response would be necessary for targeted treatment.

High incidence of toxicity and fatal adverse events were highly concerned by physicians. Dizziness and/or lethargy, constipation, abdominal pain and/or vomiting and/or nausea are most commonly seen at the beginning of treatment. Most of the symptoms relieved spontaneously without withdraw. However, there were cases that presented severe adverse events, including central venous thrombosis, and seizure, which led to hospitalization and withdraw of thalidomide. Thalidomide-induced stroke in a child with thalassemia major was also reported before (Gunaseelan and Prakash, 2017). This study still lacked of long term observation for persistent application. Neither did other previous studies. Long term adverse effects, such as pulmonary hypertension, growth retardation, fetal malformation, were not included in this report. So, the safety of long term application of low dose thalidomide in β-thalassemia children is not guaranteed and further prospective study of long term application may provide evidence on this issue.

## Conclusion

We identify that the dose of thalidomide between 2.5 mg·kg-1·d-1 and 3.6 mg·kg-1·d-1 is effective in 67.5–80.5% children with TDT, which is the first available protocol in this indication. Dizziness, lethargy, constipation, abdominal pain, vomiting, and nausea are most commonly seen, but most of the symptoms relieve spontaneously. Severe side effects are uncommon. Moreover, HbF concentration of 47.298 g·L-1 at the third month is recommended as the predictor for further major responders.

## Data Availability

The datasets used and/or analyzed during the current study are available from the corresponding author on reasonable request.
